# Functionally Graded Materials and Structures: Unified Approach by Optimal Design, Metal Additive Manufacturing, and Image-Based Characterization

**DOI:** 10.3390/ma17184545

**Published:** 2024-09-16

**Authors:** Rui F. Silva, Pedro G. Coelho, Carolina V. Gustavo, Cláudia J. Almeida, Francisco Werley Cipriano Farias, Valdemar R. Duarte, José Xavier, Marcos B. Esteves, Fábio M. Conde, Filipa G. Cunha, Telmo G. Santos

**Affiliations:** 1 UNIDEMI, Department of Mechanical and Industrial Engineering, NOVA School of Science and Technology, Universidade NOVA de Lisboa, 2829-516 Caparica, Portugal; pgc@fct.unl.pt (P.G.C.); c.gustavo@campus.fct.unl.pt (C.V.G.); csj.almeida@campus.fct.unl.pt (C.J.A.); werleyfarias@metalmat.ufrj.br (F.W.C.F.); jmc.xavier@fct.unl.pt (J.X.); mb.esteves@campus.fct.unl.pt (M.B.E.); f.conde@fct.unl.pt (F.M.C.); fa.cunha@campus.fct.unl.pt (F.G.C.); telmo.santos@fct.unl.pt (T.G.S.); 2 IDMEC, Instituto Superior Técnico, Universidade de Lisboa, Av. Rovisco Pais 1, 1049-001 Lisbon, Portugal; 3 Intelligent Systems Associate Laboratory, LASI, 4800-058 Guimarães, Portugal

**Keywords:** metals, Functionally Graded Materials, additive manufacturing, Wire-Arc Additive Manufacturing, topology optimization

## Abstract

Functionally Graded Materials (FGMs) can outperform their homogeneous counterparts. Advances in digitalization technologies, mainly additive manufacturing, have enabled the synthesis of materials with tailored properties and functionalities. Joining dissimilar metals to attain compositional grading is a relatively unexplored research area and holds great promise for engineering applications. Metallurgical challenges may arise; thus, a theoretical critical analysis is presented in this paper. A multidisciplinary methodology is proposed here to unify optimal design, multi-feed Wire-Arc Additive Manufacturing (WAAM), and image-based characterization methods to create structure-specific oriented FGM parts. Topology optimization is used to design FGMs. A beam under pure bending is used to explore the layer-wise FGM concept, which is also analytically validated. The challenges, limitations, and role of WAAM in creating FGM parts are discussed, along with the importance of numerical validation using full-field deformation data. As a result, a conceptual FGM engineering workflow is proposed at this stage, enabling digital data conversion regarding geometry and compositional grading. This is a step forward in processing in silico data, with a view to experimentally producing parts in future. An optimized FGM beam, revealing an optimal layout and a property gradient from iron to copper along the build direction (bottom–up) that significantly reduces the normal pure bending stresses (by 26%), is used as a case study to validate the proposed digital workflow.

## 1. Introduction

Historically, the concept of Functionally Graded Materials (FGMs) for engineering applications was introduced in 1984 by a group of materials scientists in the Sendai area of Japan, as a means of preparing thermal barrier materials [1]. FGMs represent a novel class of advanced composite materials in which the compositions/constituents and/or microstructures gradually change along single or multiple spatial directions, resulting in gradual changes in properties and functions, which can be tailored for enhanced performance. For example, in bonding dissimilar materials, computational simulations have shown that FGM solutions can improve the previously used composite materials because the sharp interfaces at joints can be replaced by gradient interfaces [2,3]. Consequently, a smooth transition of properties from one material to the other is obtained, thus eliminating interface problems like stress concentrations and poor adhesion [4]. The FGMs’ essential properties have made them the best candidates in several engineering fields [5], such as aerospace [6,7], automobiles [8,9], biomedicine [10,11], and defense [12,13]. It is no wonder that FGM engineering solutions are attractive, as nature itself reveals efficient designs on account of compositional grading (e.g., bones, bamboo, hedgehog spines, coral) [14]. However, it is worth mentioning that metal additive manufacturing (AM) to create FGMs raises important metallurgical concerns, such as hot cracks and intermetallic formation, which have motivated increasing research work, especially in the last 10 years [15].

In general, the various categories of FGMs include (1) porosity gradient, (2) composition gradient, and (3) microstructural gradient materials [16]. Moreover, some FGMs are designed as stepwise-graded structures, while some are designed as continuous-graded structures, depending on the field of application [17,18]. Furthermore, the versatility (or freedom) of FGM designs makes them “perfect” candidates for optimization problems [19]. In turn, topology optimization (TO) [20] has been integrated in the FGM design workflow due its higher design freedom [21]. However, TO’s results are not always AM-friendly, as manufacturability issues may arise, leading to the production of suboptimal parts [22]. Efforts have been made to improve this, addressing the paradigm of Design for Additive Manufacturing (DfAM) [23,24]. The level of complexity is particularly high in the frame of Functionally Graded Additive Manufacturing (FGAM), where layer-by-layer fabrication gradually alters the material composition and organization within a component in order to obtain the desired functionality [25,26].

The compliance minimization problem is the most frequently addressed in the literature of TO involving FGMs [27,28,29,30,31,32,33,34,35]. However, it often lacks appeal for engineering practice, which is typically focused on preventing structural failure. From this viewpoint, only a few studies have pursued strength-oriented design problems as a breakthrough in the synergy of FGMs and TO, as follows: Stump et al. [36] designed functionally graded structures using stress constraints and density-based TO. The aim was to minimize the amount of one material phase under a global von Mises stress constraint (P-norm). Conlan-Smith and James [37] solved the stress minimization of an L-bracket problem by taking advantage of FGMs to produce compliant mechanism designs that were less susceptible to failure. The authors applied a SIMP-based method, where topology and local element material properties were optimized simultaneously. Tamijani [38] carried out a density-based TO framework to optimize the spatial distribution of different materials (material composition of the structure), their interfaces, and the structural layout, achieving strength and compliance designs for multi-material structures subjected to thermal and mechanical loads. Conde et al. [39] performed multi-material TO, seeking discrete multi-material and FGM microstructures that were optimal in terms of stress distribution. To correctly model the FGM microstructures, the authors considered a multi-material SIMP interpolation scheme with specific penalty values for the mixture of materials. More recently, Silva et al. [40] solved the maximum von Mises stress minimization problem inside a design domain, through topology changes and material selection, in order to exploit the stress mitigation potential of FGMs on account of the optimal design of material composition distribution. The authors showed that a higher difference in Young’s modulus between the base materials leads to lower peak stresses.

Advances in AM processes are enabling an alternative to the material phase design approach, where materials can be continuously graded from one phase to another. In fact, multi-material AM systems, with the ability to vary material compositions locally during the building process, show great potential for FGM applications. The focus on metallic feedstocks for AM, instead of polymers, is quite a relevant topic to address. One must bear in mind that, for instance, steel is of high interest for AM systems, as it remains the most common structural material to date. Additionally, alloy-based materials satisfy typical requirements of general-purpose structural applications [41]. Among the various AM technologies, Wire-Arc Additive Manufacturing (WAAM) is one of the most suitable for producing metallic FGMs for structural applications, as it allows the use of a wide range of different materials. Consequently, by changing the composition, the range of properties (e.g., Young’s modulus, yield stress, ductility) along the component can be significantly broadened. Although there are currently several studies on the production of FGMs by WAAM, most of them focus on the transition of stainless steels to nickel superalloys since, among the possible combinations, the combination of iron and nickel is one of the least challenging to produce with high solubility between the materials and, at the same time, there is a high potential for applications in the aeronautics and aerospace industries, justifying the work on these combinations [42,43,44,45,46]. The transition from 316 L stainless steel to Inconel 625 was studied by Sasikumar et al. [42] in an abrupt material transition in the vertical direction using the Gas Metal Arc Welding (GMAW) technique, while Khan et al. [43] studied the deposition of a continuous-graded FGM and sandwich FGM using the Gas Tungsten Arc Welding (GTAW) technique. Both studies achieved defect-free deposition with a gradual transition in the interphase zones verified by EDS elemental maps, which showed uniform and gradual dispersion of the elements. Li et al. [44] studied the transition from 308 L stainless steel to Inconel 625 using the GTAW technique, where the composition gradient was obtained in the direction of the torch’s movement (horizontal), identifying the region with around 15 at.% Inconel as the weak point of the transition. Shen et al. [47] produced an iron–aluminum wall structure with a functional aluminum composition gradient from 0 at.% to over 50 at.% using the GTAW technique. It was demonstrated that the thin wall fabricated contained a chemical composition that was homogeneous in the transverse direction but less consistent both in the region affected by dilution at the bottom of the wall and in the final layers on the top surface. In addition, there have been studies into the production of abrupt transitions between the materials of stainless steel and copper [48], steel and CuAl8 [49], and 904 L stainless steel and Hastelloy C-276 [50].

The relevant contributions in the literature regarding FGM design with varying compositions using TO, as well as AM oriented towards FGM metal parts, have been reviewed above. Clearly, stress-based multi-material TO, extended to FGM design solutions, has barely been explored in the literature. Furthermore, although FGAM has great potential, real commercial applications are still very few and far between, as the related research is still in its infancy [21]. This background motivates the present work. Therefore, we propose a multidisciplinary methodology for the systematic design and fabrication of structure-specific FGM parts using a metal–metal binary system. The focus on metals here has to do with the fact that functional grading in purely metallic parts has received little attention in contrast to metal–ceramic and polymer-based FGMs. This is largely due to the comparatively slow development of viable fabrication methods. Also, joining dissimilar metals may raise important metallurgical issues, as discussed later. The proposed methodology integrates computational design and optimization, multi-feed WAAM, and image-based characterization based on full-field deformation measurements. The idea of integrating these emerging technologies into a complete thread promises to disrupt design and manufacturing workflows throughout the value chain, enabling efficiency and productivity transformation while unlocking completely new design freedom. However, due to the early stage of this specific research field, this work contributes more to concepts, rather than closing an overall engineering cycle showcasing a final produced FGM part. In fact, it raises awareness that much more research will be needed ahead for the proper completion of such a challenging engineering cycle. The results generated in this framework are based on numerical simulations and analytical modeling in order to elaborate on the layer-wise FGM concept and to pave the way for future experimental validation. For now, a key goal is to unify the TO design workflow with the WAAM process, followed by Digital Image Correlation (DIC). The proposed workflow encompasses digital data conversion of in silico models such that they can be translated into physical parts in the near future. As a proof-of-concept, the authors propose a beam design example to test the proposed digital workflow in terms of contour smoothing and the mapping of properties, which have not been covered in the literature so far. By spatially varying the volumetric composition of two metallic constituents, this numerical beam design approach also aims to leverage the advantages of FGMs over homogeneous beams, i.e., the beam’s maximum stress related to pure bending can be significantly reduced on account of an optimal Young’s modulus gradient and layout changes. Since this example has not yet been covered in the literature, the authors validate the numerical results by resorting to an analytical model, which also conveniently allows for proper generalization of the results.

In a nutshell, the novel aspects of the current work are as follows: (1) the idea of unifying TO, WAAM, and DIC to produce metal FGM parts, further discussing each of these emerging technologies individually; (2) theoretical analysis of metal–metal binary systems regarding their viability to produce FGMs; (3) optimal numerical design of an FGM beam, changing the composition and layout concurrently; (4) a beam analysis model developed to validate the obtained numerical beam results; and (5) a tested digital workflow bridging TO with WAAM by applying contour smoothing and mapping of properties to the obtained FGM design.

The rest of this paper is structured as follows: Section 2 includes critical analyses of metal–metal binary systems potentially used to produce FGM parts, as well as the variation in the Young’s modulus of the FGM with composition. Section 3 poses FGM design as an optimal design problem, including layout and material selection changes. An FGM beam under pure bending, optimized for minimum peak stress, is numerically and analytically addressed as a proof-of-concept. Section 4 addresses metal AM, mainly focusing on WAAM’s ability to produce FGMs, resorting to double-wire systems. Section 5 elaborates on full-field identification methods for FGMs. Section 6 presents a workflow for digital data processing, connecting the design and manufacturing stages. Section 7 discusses the current authors’ contribution by comparing it with other relevant published works in the field. Finally, Section 8 presents the conclusions and future trends.

## 2. Material Compositions

### 2.1. Metal–Metal Binary Systems

For the design of functionally graded additively manufactured metal parts, it is necessary to consider metal–metal binary systems, realizing that a binary choice must ponder several aspects. On the one hand, selectively placing dissimilar metals has long been a strategy for meeting spatially varying service demands. Table 1 presents a variety of pure metals, and their respective Young’s moduli, for a non-oriented and fine-grained material (e.g., wrought or hot-rolled alloys). Every combination among these metals is highlighted in terms of Young’s modulus ratios (see the upper triangular part of Table 1). The more dissimilar the moduli, the greater the potential of the resulting FGM to minimize the maximum von Mises stress, as shown in Section 3 on account of numerically finding an optimal Young’s modulus gradient over a design domain. Unfortunately, a metal–metal pair choice may not be so obvious because, on the other hand, some binary systems are considered to be unweldable (or unprintable). Just a few binary systems are characterized by complete solubility over all compositions and temperatures. In fact, the formation of brittle intermetallic phases and hot cracks is without question the biggest challenge encountered when joining dissimilar metals. Other dissimilar joining issues include thermal property mismatch and other metallurgical effects [26]. Some of these issues are discussed below and summarized in Table 1 (see the lower triangular portion).

**Figure 1:**
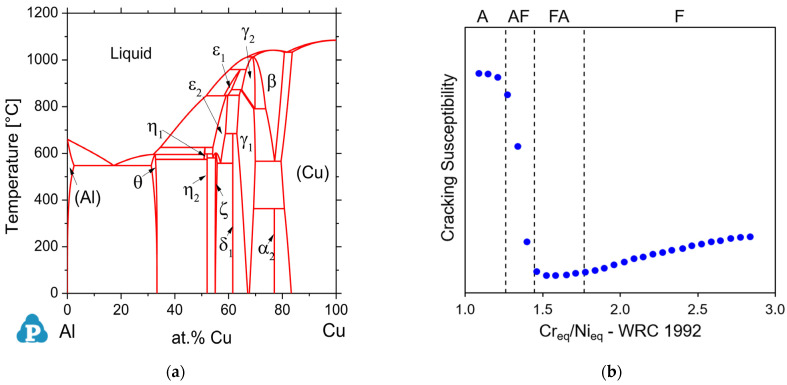
(**a**) Experimental and simulated Al-Cu phase diagram [59]. (**b**) Influence of Ni equivalents on hot crack susceptibility. A, AF, FA, and F denote transitions in solidification modes (austenitic, austenitic–ferritic, ferritic–austenitic, and ferritic, respectively), depending on the composition. The blue circles indicate the susceptibility to hot cracking. As susceptibility increases, the weldability and printability of the alloys decrease.

To exemplify the problems mentioned in the above paragraph, the authors used the Al-Cu phase diagram (intermetallic formation; see Figure 1a) and the weld solidification crack susceptibility as a function of composition for stainless steels (see Figure 1b). For instance, Figure 1a indicates that Al alloys (e.g., 2xxx series) have a significant solubility of Cu (about 5%). Therefore, during solidification, the matrix (α–Al-rich) is formed first, and the Cu content increases in the remaining liquid (interdendritic enrichment), which promotes the eutectic reaction (L→α+θ) in the remaining interdendritic liquid [51]. Thus, the final microstructure of Al 2xxx series is characterized by a dendritic microstructure and eutectics in the interdendritic region. Looking now to the other side of the stable diagram (Cu-rich side), it can be observed that Cu has a higher solubility of Al (about 15%), which can range from pure Cu alloys to commercial alloys like CuAl8 (AWS A5.7 ERCuAl-A1) [52]. These alloys start their solidification from a Cu-rich phase, and some minor elements segregate from the liquid, which can induce a eutectic reaction (L → Cu+β).

One point that must be highlighted is that the above-cited alloys (e.g., Al 2xxx series and CuAl8) can be manufactured via fusion-based AM processes, such as PBF and DED. However, when attempting to mix them to generate an FGM, some metallurgical problems can arise, depending on the dilution level, i.e., the type of AM process. For dilution levels between approximately 6% and 85% Cu, a large quantity of deleterious intermetallics can form during solidification and/or solid-state phase transformations, making it nearly infeasible to join Al-rich and Cu-rich alloys via fusion-based processes, especially those with high dilution, such as laser and arc plasma DED. Also noteworthy is the fact that similar issues can occur when attempting to join commercial alloys related to Fe-Al [53] (e.g., low-alloy steel and Al 2xxx series), Ti-Al [54,55] (e.g., Ti-6V-4Al and Al 7xxx series), Ni-Al (e.g., Inconel 718 and Al 2xxx series), and Fe-Ti [56] (e.g., stainless steel and Ti-6V-4Al) systems. Despite the fact that individual alloys can be fabricated via fusion-based processes, the chemical composition of the “intermediate” alloy induces the formation of several intermetallics, which can compromise the fabrication and related performance of FGMs.

In addition, certain alloy systems (e.g., Fe-Ni) exhibit a wide dissolution range, facilitating the mixture of their commercial alloys at different dilution levels (e.g., welding low-alloy steels with Ni-based filler metals). Consequently, these alloy systems generally do not experience significant formation of deleterious intermetallics. Instead, the primary concern in FGMs relates to hot cracks, including issues during solidification and liquation, as shown in Figure 1b. An increase in the Ni equivalent (WRC-1992) changes the solidification mode, increasing susceptibility to hot cracks. This is why FGM deposition is typically not observed in dissimilar welded joints [57,58].

Moreover, alloys fabricated via AM typically exhibit high residual stresses, necessitating post-deposition heat treatments [60,61]. This poses a significant challenge for FGMs, as heat treatments (e.g., peak temperature and soaking time) are customized for specific alloys. Thus, for a multi-alloy component like an FGM, heat treatments may potentially compromise the mechanical properties on certain sides of the FGM component. Manufacturers must carefully select which material side to prioritize such that this effect is minimized [62,63]. This challenge becomes more critical for precipitation-strengthened alloys, such as the Al 2xxx series and Inconel 718, which require extended aging heat treatments (e.g., approximately 18 h) within narrow temperature ranges [64]. For example, creating an FGM using low-alloy steels and Inconel 718, which exhibits high solubility in the Fe-Ni system, is feasible due to their good metallurgical compatibly and resistance to hot cracks. However, achieving a heat treatment that does not compromise any side of the FGM component is nearly impossible. To the best of the authors’ knowledge, this has not yet been achieved. Therefore, while FGMs may present a viable alternative for numerous applications, manufacturing them via fusion-based AM processes poses challenges that require a thorough understanding of the AM process–material relationship, particularly during the component design.

In summary, despite Table 1 indicating several material combinations (66 possible systems), the practical number of viable FGM systems decreases significantly when considering factors such as the limited availability of commercial alloys, alloy compatibility, susceptibility to hot cracks, and the lack of suitable heat treatments. In this context, the lower triangular portion of Table 1 highlights possible mixtures between pure metals, along with their primary anticipated challenges based on data provided by the phase diagrams from Thermo-Calc^®^. It is also important to note that the analysis carried out here is superficial and serves only as a guide, highlighting potential difficulties that may arise when attempting to print FGMs using two specific metal systems and their respective alloys.

### 2.2. Library of Compositions and Properties

**Figure 2:**
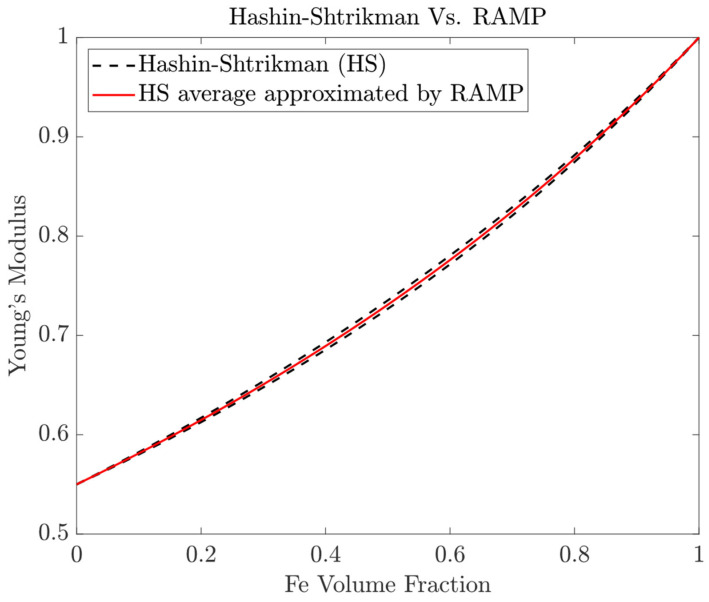
Theoretical variation in the Young’s modulus for the Fe-Cu system based on the HS bounds.

To obtain specific oriented FGM metal parts, it is necessary to generate data regarding the mechanical properties of material compositions. A large enough spectrum of material compositions must be selected to generate statistically representative data. Eventually, this strategy mitigates the risks of not being successful in homogenizing some material composition in practice via the AM process. Therefore, macroscopically composition-targeted samples can be created using the multi-feed wire variant of the WAAM process. The objective is to produce samples with different chemical compositions that will map the design space of mechanical properties. Different pure materials, as covered in Section 2.1, could be selected to create graded samples. In the present framework, the binary system Fe-Cu from Table 1 is selected as an example. Different material compositions can be created to measure the evolution of reference mechanical properties, namely, the Young’s modulus as a function of the following pre-selected volume fractions of Cu: 0%, 20%, 40%, 60%, 80%, and 100%. Several specimens per material composition might be manufactured and then characterized, as further discussed in Section 4. The resulting data can then be processed and translated to a suitable library of material compositions and mechanical properties. Furthermore, an interpolation of such experimental data to obtain a continuous mapping between volume fractions and properties is of great value as an input for the computational design of FGMs. However, in view of the absence of such experimental data, the compositional gradient predictions based on the Hashin–Shtrikman (HS) bounds [39,40,65] can be used as an alternative. Figure 2 plots such lower and upper bounds with dashed lines, considering the Fe-Cu system with a Young’s modulus ratio equal to 0.55 according to Table 1. The present framework thus assumes a continuous variation in Young’s modulus between 0.55 and 1 (the property ratio is used instead of absolute values), following the trend given by the average of the HS bounds (solid line, RAMP [66], as detailed in the next section) along the highlighted 0.2 intervals of the Fe volume fraction (the complement is the Cu volume fraction). The FGM results shown later in this work are thus based on this theoretical curve. In future work, an experimental curve should be obtained to replace the current one for feeding predictive computational FGM design models.

## 3. Optimal Design

The FGM (or graded structure) design problem can take advantage of TO. In this section, the proposed approach involves obtaining a compositional grading as an optimal material selection problem, along with optimal changes in structural layout. For example, we consider the design problem of an FGM beam as a follow-up on the authors’ previous work [40], which readers should consult for a thorough understanding of the material model and design methods used, which are only briefly described below.

### 3.1. Numerical Model

**Figure 3:**
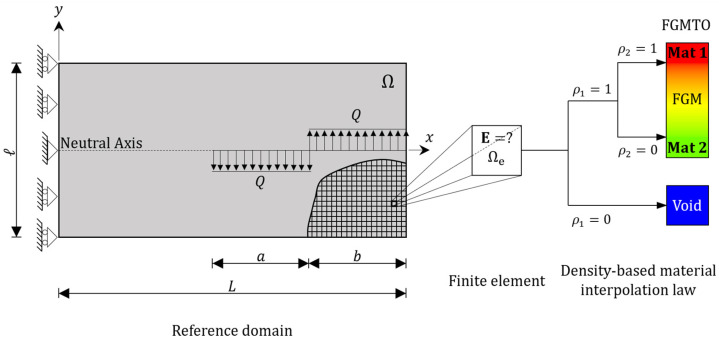
Two-dimensional (half) beam numerical model. The distributed loads Q generate a torque such that the beam is subject to pure bending. Representation of the FGMTO density-based material interpolation scheme.

The numerical model presented in this section is a two-dimensional beam (reference domain Ω), which is discretized using a regular Finite Element (FE) mesh (see Figure 3). Only half of the beam is modeled, due to symmetry. To generate a torque such that the beam is subject to pure bending, one must apply opposite distributed loads (Q), which also prevent highly localized stresses. Furthermore, a density-based approach is used here to solve the discretized TO problem with continuous design variables [20,66]. Therefore, to each FE, we assign an isotropic stiffness tensor (E) that depends on the design variables (densities) though a bi-material interpolation scheme, as detailed below, at a given point in Ω. Isotropic symmetry is considered here as a preliminary design approach, despite the known fact that the layer-by-layer printing process introduces directionality in the resulting material properties. This should obviously be investigated once experimental results are available, which may require proper adaptation of the material model to reflect orthotropy symmetry instead, e.g., [67,68]. All numerical data shown below were handled by the open-source FEM 2D Fortran code [40], as well as the post-processor of the ANSYS^®^ research license. Plane stress was assumed.

Since the goal of the proposed framework is the concurrent optimization of the topology (solid–void) and the material composition distribution by mixing two homogeneous materials (Mat 1 ≡ Fe and Mat 2 ≡ Cu) in the beam’s domain to generate an FGM, a density-based approach is pursued as follows: The design variables are (1) the distribution of solid ($\rho_{1}\in [\rho_{min},1]$) in the reference domain and (2) the volume fraction of Mat 1 in the solid region ($\rho_{2}\in [\rho_{min},1]$). To avoid singularity issues, a small positive value is assumed here for the lower bound on both design variables ($\rho_{1}$ and $\rho_{2}$), i.e., $\rho_{min}=10^{-3}$. Additionally, to avoid numerical problems such as checkerboard patterns and mesh dependence, a density-based filtering technique is used [69]. This technique is applied to $\rho_{1}$ design variables only to obtain the respective filtered densities ${\tilde{\rho }}_{1}$. The original design variables $\rho_{1}$ have no physical meaning. The material selection design variables, $\rho_{2}$, are not filtered in this work. Both $\rho_{1}$ and $\rho_{2}$ are assumed to be uniform within each FE. Regarding the stress fields, the density filter also promotes smoothness on the stress distributions at boundaries between phases (solid–void), preventing singularities at jagged boundaries (unphysical stresses).

Essentially, an FGM beam design solution mixing two homogeneous solids (Mat 1 and Mat 2) is sought in the present work. To solve the density-based TO problem by taking advantage of an FGM (the FGMTO problem, as schematically represented in Figure 3), both SIMP and RAMP interpolation schemes can be used [40]. The SIMP scheme is applied for the purpose of layout definition, whereas the RAMP scheme is used to model the rule of mixtures in FGMTO. Therefore, the density-based multi-material interpolation law found in [40] is used here, i.e.,

(1) $$E={{(\rho }_{1})}^{p}\left[\frac{\rho_{2}}{1+{q(1-\rho }_{2})}E_{1}+\left(1-\frac{\rho_{2}}{1+{q(1-\rho }_{2})}\right)E_{2}\right],$$

where the artificial densities $\rho_{1}$ and $\rho_{2}$ are within the bounds defined above, $E_{1}$ and $E_{2}$ are the stiffness tensors of the solid phases (with $E_{1}>E_{2}$), and p and q are the SIMP and RAMP penalization parameters, respectively. Typically, p is greater than 1, so intermediate densities are unfavorable in compliance-based problems (typically p ≥ 3 in 2D problems [20]). As regards the value of q, it must be carefully chosen to accommodate the desired penalization effects and consistency with the physics of solid mixtures. In this work, intermediate values of $\rho_{2}$, which set the proportion of each solid phase (Mat 1 and Mat 2) in the resulting solid mixture, must be consistent with the bounds provided for the effective elastic moduli of multiphase materials, e.g., the HS bounds [65]. These are upper and lower bounds for the elastic moduli of composite materials depending on the volume fractions of each constituent material, and it is assumed that these materials are mixed uniformly, with no microstructure. Assuming the 2D case and that both materials are isotropic, as is their mixture, with the same Poisson’s ratio (equal to 1/3), the HS bounds can be expressed as follows [29,70]:

(2) $$E_{HS}^{u}=\frac{\rho_{2}E_{1}+(3-\rho_{2})E_{2}}{\left(3-2\rho_{2}\right)E_{1}+2\rho_{2}E_{2}}E_{1},$$

(3) $$E_{HS}^{l}=\frac{(2+\rho_{2})E_{1}+(1-\rho_{2})E_{2}}{2\left(1-\rho_{2}\right)E_{1}+(1+2\rho_{2})E_{2}}E_{2},$$

where $E_{HS}^{l}$ and $E_{HS}^{u}$ are the lower and upper HS bounds, respectively. Therefore, for the RAMP interpolation scheme in Equation (1), assuming $\rho_{1}=1$, to be within the HS bounds, one should choose $q=2(E_{1}-E_{2})(E_{1}+5E_{2})/6E_{2}\left(E_{1}+2E_{2}\right)$ for materials with a Poisson’s ratio equal to 1/3 [66]. Based on this q expression, one realizes that the parameter q is material-dependent. For example, selecting the Fe-Cu binary system from Table 1, it gives q=0.487, meaning that the resulting RAMP curve closely approximates the average of the HS bounds (see Figure 2).

### 3.2. Problem Formulation

The aim is to bring the maximum effective stress (e.g., the von Misses stress) in the beam sketched in Figure 3 to a minimum. However, a min–max problem raises non-differentiability issues. To tackle this, the maximum von Mises stress might be replaced by a differential (smooth) approximate function, such as the Kreisselmeier–Steinhauser, P-norm, or P-mean (so-called aggregation functions in the literature to reduce the number of stress constraints) [71,72]. Alternatively, in order to alleviate the inherent difficulty of non-differentiability, the so-called “bound formulation”, as suggested by Taylor and Bendsøe [73], is adopted here (see also [39,40,74]). As seen below in Equation (4a), this means replacing the original min–max stress problem with the problem of minimizing a variable z subject to stress constraints bounded by z, i.e., $\sigma_{e}^{VM}<z$. The bound z is an additional design variable that replaces the non-differentiable functional, max$\sigma_{e}^{VM}$, and z∈ ]0,+∞[.

(4a) $$\underset{\rho_{1},\rho_{2},z}{\min}z,\;\rho_{min}\leq \rho_{1,i}\leq 1;\;\rho_{min}\;\leq \rho_{2,i}\leq 1;\;\rho_{min}=10^{-3};\;i=1,\ldots ,N,\;{\tilde{\rho }}_{i}\left(\rho \right)=\frac{\sum_{j\in N_{i}}\omega \left(y_{j}\right)\rho_{j}}{\sum_{j\in N_{i}}\omega \left(y_{j}\right)};\;\omega \left(y_{j}\right)=max\left\{0,R_{min}-\left\|y_{j}-y_{i}\right\|\right\},$$

(4b) $$s.t.\frac{\sigma_{e}^{VM}(u\left({\tilde{\rho }}_{1},\rho_{2}\right),{\tilde{\rho }}_{1}{,\rho }_{2})}{z}\leq 1,\;e=1,\ldots ,N,$$

(4c) $$\frac{C\left(u\left({\tilde{\rho }}_{1},\rho_{2}\right)\right)}{C^{*}}\leq 1,$$

(4d) $$\frac{V({\tilde{\rho }}_{1})}{V^{*}}=\frac{\sum_{i=1}^{N}\left[{\tilde{\rho }}_{1,i}\left|\Omega_{i}\right|\right]}{\sum_{i=1}^{N}\left|\Omega_{i}\right|V^{*}}\leq 1,$$

(4e) $$\frac{\varphi_{1}({\tilde{\rho }}_{1})}{\zeta_{1}}=\frac{\sum_{i=1}^{N}\left[\left(1-{\tilde{\rho }}_{1,i}\right)\left({\tilde{\rho }}_{1,i}-\rho_{min}\right)\right]}{\zeta_{1}}\leq 1.$$

In Equation (4b,c), **u** solves the equilibrium equation Ku=f; V is the volume fraction; C is the compliance of the structure; $\varphi_{1}$ measures the level of intermediate values (gray) present on the filtered density field ${\tilde{\rho }}_{1}$, with $\zeta_{1}\in \;]0,+\infty [;$ and N is the total number of elements present in the FE mesh. Furthermore, all constraints are written in the format to be read by the MMA optimizer [75]—Fortran version.

The compliance constraint in (4c) is used to ensure that the resulting design is stiff enough, i.e., connectivity of the solid phase exists, and the trivial solution of structure absence is avoided [76]. Essentially, from the SMTO compliance minimization problem, it results in a compliance value that is taken as reference to set a compliance upper bound $C^{*}$ for the FGMTO stress minimization problem.

The intermediate density values of $\rho_{2}$ have physical meaning here, as they represent different proportions of each solid (Mat 1 and Mat 2) present in the solid mixture (recall Section 3.1). However, one should realize that the exponent p in Equation (1) may not be sufficient to penalize the intermediate densities (gray) in ${\tilde{\rho }}_{1}$. Therefore, the intermediate densities constraint in (4e) is proposed to limit the amount of ${\tilde{\rho }}_{1}$ intermediate values, such that solid–void regions can be clearly identified. Bear in mind also that the averaging related to the density filter necessarily opens an exception regarding the presence of gray. Gray will always appear at the border of each two neighboring phases (solid–void). Hence, careful choice of the parameter $\zeta_{1}$ is demanded, which can be comprehensively problem-dependent. For further details, including sensitivities, the reader is referred to Silva et al. [40].

### 3.3. Numerical Results

Consider again the beam sketched in Figure 3, and assume the following data: L=2.1 m, l=0.5 m, a=L/3 m, and Q=204.082 kN/m, which generates a bending moment of 100 kN ∙ m at the fixed support located at the Neutral Surface (NS). The thickness of the beam is assumed to be unitary for the sake of simplicity. Furthermore, in this work, the eight-node isoparametric quadrilateral FE (Q8) is used, which avoids the checkerboard problem. Due to symmetry, only half of the beam is modeled, which helps reducing the number of design variables and stress constraints. All nodes along the symmetry line have their horizontal displacement constrained, but the node located at the NS also has its vertical displacement constrained to prevent rigid-body motion (see the sketch of boundary conditions in Figure 3).

Taking advantage of the information provided in Table 1, one can choose to use normalized material properties instead of using absolute values as the input data for this beam problem. Hence, considering the Fe-Cu system again, one can define the Young’s modulus $E_{1}$ of Fe as unitary, i.e., 1 GPa. Then, the Young’s modulus value for Cu, $E_{2}$, can be interpreted as being a property ratio. Below such ratio is inferior to 1, taken as 0.55 here (see Table 1). The Poisson’s ratio is assumed to remain equal to 0.3 for both Fe and Cu due to the use of the HS bounds [70]. All stress results in this section are always to be read in MPa.

In order to obtain a regular FE mesh, the beam is meshed with 126 elements in the horizontal direction and 30 elements in the vertical direction. As regards the compliance upper bound $C^{*}$ in (4c), one can make it equal to the optimal compliance obtained when solving the single-material compliance minimization problem subject to a volume fraction constraint (V=0.85) using as base material $E_{2}=0.55$ GPa, i.e., $C^{*}=1154$ J. As regards the volume fraction upper bound $V^{*}$ in Equation (4d), one can set it to 0.85 (85% solid material). In Equation (4e), the final value of $\zeta_{1}$ is 26.4. However, keep in mind that this $\zeta_{1}$ value must be tuned by running the optimization a few times such that, at the optimum, only a certain amount of “gray” remains due to the density filter between solid–void regions. The filter radius for densities ${\tilde{\rho }}_{1}$ is kept constant throughout the optimization history and only collects for averaging the eight adjacent element neighbors of each element in the FE mesh (2D problem). Regarding the material interpolation scheme (1), one can use constant penalization parameters p and q equal to 4 and 0.487, respectively, in line with the information provided in Section 3.1. Moreover, an initial uniform distribution of 0.85 is considered for $\rho_{1}$ density variables. As regards the initialization of $\rho_{2}$, uniform density values equal to 0.5 are used, with exceptions made for the beam’s extreme fibers, where $\rho_{2}=\rho_{min}$.

Interestingly, in the example considered in this work, the von Mises stress and densities are equal at opposite elements facing the NS (the NS and the beam’s centroid are coincident), which means that the number of design variables and stress constraints can be halved, as design variables are coupled in pairs. Such a simplification requires stress sensitivities to be adjusted, because elements at opposite sides of the NS share the same density variable.

**Figure 4:**
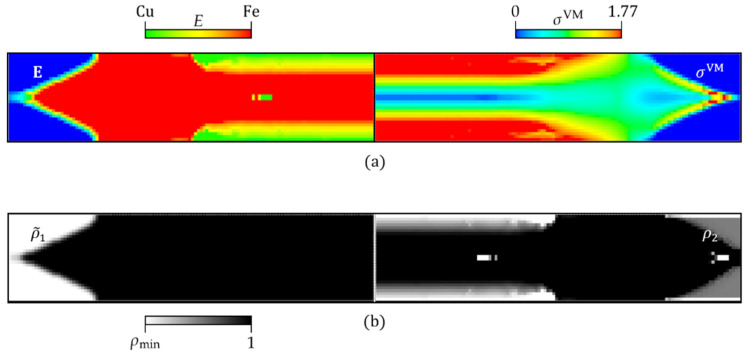
FGM result for the beam example solving the optimization problem (4): (**a**) Young’s modulus distribution (**left**) and von Mises stress map (**right**). (**b**) Density fields.

Figure 4 shows the FGMTO problem’s solution. In particular, Figure 4a illustrates the Young’s modulus distribution (left) and von Mises stress map (right) on opposite halves of the full beam’s length. Figure 4b shows the corresponding ${\tilde{\rho }}_{1}$ and $\rho_{2}$ density fields. However, recalling Equation (1), it is noteworthy that the density field $\rho_{2}$ is only meaningful in elements where ${\tilde{\rho }}_{1}=1$. The maximum peak stress is highlighted by the color scale (see Figure 4a, right-hand side). The FGM solution obtained here is a layer-by-layer gradation of properties (concept of layer-wise FGM) that is responsible for converting the original linear stress distribution of pure bending into a more uniform one. Note that, in the FGM’s region of influence, each layer has constant stress, and stress uniformity is obtained across several layers, as shown in Figure 4a (right-hand side), which reduces the original peak stress from the linear stress distribution of pure bending verified at the mid-span of the beam, i.e., the peak stress decreases from $\sigma^{VM}=My/I=2.40$ MPa (flexure formula for pure bending with M=100 kN·m; y=0.25 m; and $I=1.042\times 10^{-2}\;m^{4}$) to $\sigma^{VM}=1.77$ MPa (26% peak stress reduction). Also noteworthy is the fact that a fully stressed design cannot be attained, as stresses will always vanish at the NS. Obviously, as the FGM’s region of influence is ratio $E_{2}/E_{1}$-dependent, the higher the ratio, the smaller the gradation area and the higher the peak stress, and vice versa. More insights about this trend are given in the next section.

Based on the layer-wise FGM found here, curve fitting can be applied using a set of points obtained for Young’s modulus in the FGM’s region of influence, i.e., at the mind-span of the beam. Essentially, one arrives here to the following hyperbolic law for the variation in Young’s modulus: $E\left(y\right)=-0.1385/y$ if y∈[−0.25;−0.1385], and $E\left(y\right)=0.1385/y$ if y∈[0.1385;0.25], which perfectly matches the trend predicted by the numerical simulation, as the coefficient of determination of this curve fitting ($R^{2}$) is equal to 1.

### 3.4. Analytical Approach

The FGM beam solution presented in Section 3.3 (layer-wise FGM) provides the following insight: an optimal variation in the Young’s modulus for a beam under pure bending is hyperbolic across part of the beam’s thickness (*y*-axis), whereas, in the remaining part, the modulus remains constant, i.e., $E(y)=E_{1}$. The area of the beam influenced by the layer-wise FGM has a constant stress distribution. This obviously contrasts with the homogeneous beam case, which has a linear stress distribution along the *y*-axis, regardless of the base material.

The mainstream FGM beam analyses encompass FE and analytical approaches, often including the following two material functions: power law (P-FGM) and exponential law (E-FGM) [77,78]. However, none of these functions minimizes the beam’s normal stresses as intended here. Therefore, the mathematical formulation for an FGM beam analysis, as proposed by Althoey and Ali [77], is revisited and adapted here to accommodate the hyperbolic law variation of E(y) (see Table 2) and to validate the numerical results shown previously. The resulting normal beam stresses are calculated through Equation (5), which depends on the bending moment M and on the inverse stiffness coefficients $K^{*}$ given by Equation (6). These coefficients, in turn, depend on the stiffness coefficients given by Equation (7), which requires integration along the beam’s transverse direction. Note that, in Equation (5), the ratio $K_{2}^{*}/K_{3}^{*}$ is equal to zero and M(x)=M (constant), as the beam in Figure 3 is under pure bending.

(5) $$\sigma_{xx}\left(x,y\right)=K_{3}^{*}E\left(y\right)M\left(x\right)\left[\frac{K_{2}^{*}}{K_{3}^{*}}+y\right],$$

(6) $$K_{2}^{*}=\frac{{-K}_{2}}{K_{1}K_{3}-{K_{2}}^{2}};\;K_{3}^{*}=\frac{K_{1}}{K_{1}K_{3}-{K_{2}}^{2}},$$

(7) $$K_{1}=\int E\left(y\right)dy;\;K_{2}=\int yE\left(y\right)dy;\;K_{3}=\int y^{2}E\left(y\right)dy.$$

Since the axial stress is zero at the Neutral Axis (NA) of the FGM beam, stress uniformity is never attained (recall also Figure 4a, right-hand side). Interestingly, the portion of the FGM beam with uniform stress becomes thicker, and lower in stress magnitude, as the base materials become more dissimilar, i.e., the ratio $\hat{r}=E_{2}/E_{1}$ decreases. Here, it is assumed that Mat 1 is stiffer than Mat 2 (i.e., $E_{1}>$ $E_{2}$). The analytical maximum stress reduction (in %) achieved by an FGM beam with a hyperbolic law variation in Young’s modulus is given by $\delta =100\times \left(2/(3-{\hat{r}}^{2})-1\right).$ For example, consider the following modulus ratios: $\hat{r}=$ 0.75, 0.50, and 0.25. Therefore, the maximum stress reduction achieved by the FGM beam solution, compared to the homogeneous one, is about 18%, 28%, and 32%, respectively. In particular, for the Fe-Cu ratio, the maximum stress reduction obtained by this latter analytical expression is 26%, which validates the numerical stress reduction obtained in the previous section.

From Table 2, one can see that, as the property ratio $\hat{r}$ approaches zero ($\hat{r}\to 0$), the stress analytical term, which includes the evaluation at the NA, increases towards infinity ($\sigma_{xx}\to \infty$), i.e., $\underset{\hat{r}\to 0}{\lim}0.5y12M/{\hat{r}h}^{3}(3-{\hat{r}}^{2})⟶\infty$, which is not acceptable. In engineering practice, $\hat{r}$ is always finite and must be consistent with the materials’ compatibility, which typically implies combining two materials whose Young’s moduli do not differ significantly (recall Section 2.1).

## 4. Additive Manufacturing

The production of FGMs through conventional manufacturing processes presents several challenges, such as spatial control of the chemical composition, limits on the design of the components, adaptation of manufacturing processes to mass production and upscaling, repeatability of production processes, reliability of the produced FGMs, cost-effectiveness of production processes, and quality control. All of these limitations have hindered the widespread use of FGMs in everyday applications [79]. However, recent advanced manufacturing technologies, such as AM, have features that promise to facilitate FGMs’ production and offer new possibilities for greater control and flexibility in the change of chemical composition.

WAAM stands out from other AM processes due to its high deposition rate, which allows large parts to be produced in a useful time. For the production of FGMs by WAAM, the use of the GTAW technique stands out, where gradient functionality can be obtained through the use of a mechanism that allows the control of the feeding of different filler materials in wire form. To obtain a gradient of chemical composition, it is enough to vary the speed of each material wire to be added to the melting pool, thus allowing the deposition of different materials in specific layers, as well as changes in composition within the same layer [80].

Although it is technically possible to produce FGMs by WAAM, there are various challenges and limitations that must be considered when producing a physical component from a numerical model. The inherent characteristics of the material deposition process impose limits on spatial resolution. In the build direction (height), WAAM typically produces layers with a thickness ranging from 1 to 4 mm [81], which constrains changes in composition along the build direction to a minimum resolution equivalent to the layer height. The deposition of a new layer involves the partial fusion of the previous layer, resulting in a dilution of the material of the previous layer with that of the new layer; this is referred to as weld penetration, and it can vary from tenths of a millimeter up to 4 mm, depending on the deposition parameters [82]. Although this dilution can be controlled by adjusting the process parameters, such as the current and the wire feed speed, it cannot be entirely eliminated. This imposes limitations on the control of the resulting composition gradient, making the production of sharp gradients unfeasible. Therefore, the gradient slope can be modulated by varying the chemical composition of each layer and by managing the transition between successive layers through careful control of dilution via process parameter adjustments [43].

In the deposition direction (horizontal plane), the spatial resolution limit is dictated by the dynamics and dimensions of the melt pool, which can range in length from approximately 4 to 20 mm. This length is primarily influenced by process parameters that govern heat input, as well as the thermal conductivity of the material being deposited [82]. Consequently, within the same layer, and along the horizontal direction, there is a dilution effect where the material being deposited at any given moment mixes with the material deposited immediately prior, even with abrupt changes in material composition.

Another challenge is related to the presence of defects such as porosity, cracks, and inclusions in the components produced by WAAM, which compromise the mechanical properties. The origin of these defects can be related to various factors but is often associated with the selection of inappropriate process parameters, inadequate gas shielding, metallurgical incompatibility, or contamination of the feedstock material [83]. In particular, the optimization of the process parameters for each chemical composition of the deposited material plays a fundamental role in the production of FGMs. As the chemical composition varies along the geometry, the probability of defects increases due to the difficulty in maintaining the parameters appropriate for the composition being deposited, thus requiring them to be dynamically adapted to these variations.

In addition, the order in which the different materials are deposited can also be a limitation, since the deposition of a material with a higher melting temperature on a material with a lower melting temperature can lead to the collapse of the component, while the opposite is possible. This occurs because the thermal energy required to melt the high-melting-point material may exceed the capacity of the underlying material to dissipate the heat, causing it to surpass its melting temperature and subsequently collapse.

Finally, the thermal cycles to which the component is subjected during its WAAM production can also be a limitation to the production of FGMs, since they generate residual stresses due to the different heating and cooling rates within the part, leading to distortions and potential cracking [84]. In an FGM, the gradient of material properties, such as the coefficient of thermal expansion, can aggravate the formation of residual stresses, since the material with the highest coefficient of thermal expansion will have greater displacements and cause plastic deformation of the adjacent material that has a lower thermal expansion coefficient. These thermal cycles in WAAM are also known to affect the materials’ microstructure and produce a coarse-oriented microstructure, resulting in anisotropic properties in most materials. Thus, the final properties of the component are dependent not only on the volumetric fraction of the deposited materials but also on the thermal history during deposition.

**Figure 5:**
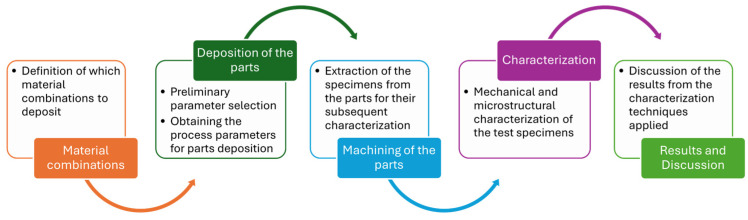
Suggested experimental workflow to address challenges when producing an FGM by WAAM.

Therefore, in this framework, and in order to address some of the challenges that arise during the production of an FGM by WAAM, the experimental workflow of Figure 5 is suggested. The procedure starts by defining which combinations of metallic materials can be deposited by ensuring the materials’ structural compatibility. Secondly, it is necessary to properly adjust the process parameters (i.e., arc voltage, electric current, travel speed, wire feed speed, etc.) for each material composition to be deposited. Thirdly, to perform a deep characterization of each composition, the specimens need to be extracted from the parts. Lastly, for the mechanical and microstructural characterization of the deposited materials, the following characterization techniques can be applied: visual inspection for the detection of major defects such as cracks; microstructure analysis, Vickers microhardness measurements, and electrical conductivity measurements to ensure material homogeneity; uniaxial tensile tests, fractography, and dilatometry tests to assess mechanical properties and thermal expansion coefficients.

## 5. Image-Based Characterization

The gradual achievements in computational design assisted by TO, along with the development of the WAAM process for material compositions and FGM structures, will pave the way toward a new layer of integration based on full-field deformation data. On the one hand, DIC will assist in the mechanical characterization of individual material compositions and FGM parts manufactured by WAAM [85]. On the other hand, the DIC procedure will be leveraged at the simulation stage in order to create a virtualization platform for product design, fully consistent with the spatial resolution and uncertainties that are typically observed experimentally [86,87,88].

Tensile mechanical tests on composition-targeted samples are assisted by full-field deformation measurements provided by DIC. The isotropy and uniformity of the displacement and strain fields under loading is assessed. Suitable specimen preparation in terms of speckle-pattern isotropy and quality are guaranteed. Moreover, parametric analyses are carried out to select the DIC setting parameters (e.g., subset size, subset step, correlation criterion, shape function, image interpolation and pre-filtering, strain window size, strain tensor metric, and displacement-to-strain interpolation algorithm) in a suitable balance between camera-based resolution and accuracy. Since samples produced by WAAM are homogenized over target material compositions, mechanical properties are directly determined from closed-form solutions (linking material properties to strain, dimensions, and resultant loading measurements). These explicit equations are mathematically derived from mechanical models under the assumption of a homogeneous stress state and Saint-Venant’s principle.

Moreover, DIC measurements can be integrated across the workflow, dealing with simulation-driven product development, in which a simulation and experimentation are fully integrated as a part of the mechanical design of FGM parts. A digital twin of the mechanical state of the FGM parts can be developed with the assistance of full-field data. An inverse strategy can then be implemented to determine the spatial variability of mechanical properties in order to validate the design and manufacture procedure, i.e., in terms of spatial resolution. Consequently, image-based deformation measurements can be fully integrated in the computational design workflow for validation purposes.

## 6. Digital Data Processing Workflow

**Figure 6:**
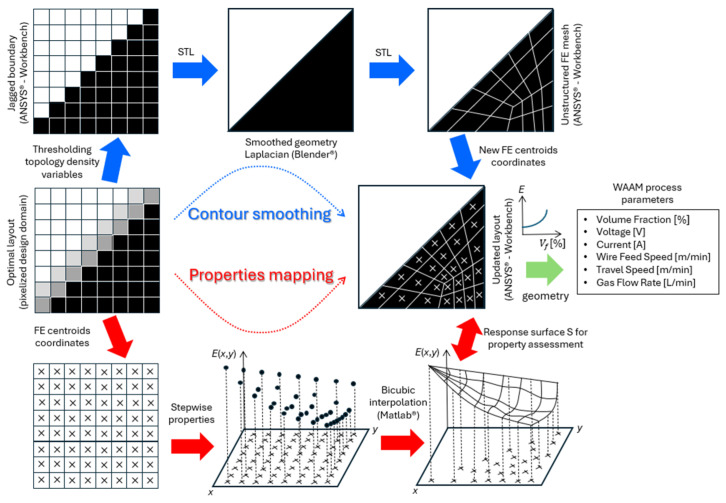
Digital workflow converting digital data from TO to the AM system.

Density-based TO describes a structure by a “raster representation” of gray scales, where gray is interpreted as an artificial density of the material, varying between 0 and 1. Gray is penalized along iterations by means of a density interpolation scheme (e.g., SIMP, RAMP), such that clear black-and-white end designs are obtained. However, jagged boundaries may appear during the optimization process [20], which requires proper post-processing in view of fabrication. Hence, a digital workflow is proposed here to translate the optimal design, on top of a regular FE mesh, into one adapted to be more AM-friendly. In the context of optimized structures made of FGMs, the proposed workflow comprises two main procedures: (1) contour smoothing, and (2) mapping of properties. In a nutshell, the former generates a manufacturable design by applying boundary smoothing. Today, this is a standard post-processing procedure based on the Laplacian operator. However, in the case of FGM designs, this procedure overlooks data transfer related to the optimal distribution of material properties. These data are tied to the original mesh, used for optimization, and cannot be directly transferred to the smoothed structure, requiring a second procedure. The latter essentially interpolates the original properties database to generate a surrogate model. These two procedures are combined in the workflow shown in Figure 6 and are detailed below.

Typically, in ANSYS Mechanical APDL^®^, a square-grid mesh of plane elements discretizes the design domain, wherein the optimal structural topology is defined by the stepwise distribution of $\rho_{1}$ densities (see Figure 6, center-left). Importantly, the void and solid identifications are based upon a threshold applied to $\rho_{1}$ densities, i.e., one considers solid regions for densities ≥0.5 and void regions for densities <0.5. The 0.5 threshold value is considered to be fair here, as $\rho_{1}$ densities are filtered according to the filtering technique formula proposed by Bruns and Tortorelli [69], which results thereafter in structures with blurred contours, where the $\rho_{1}$ densities range between 0 and 1. A valid exception to the 0.5 threshold applies to the low-density region ($\rho_{1}\ll 0.5)$ observed at the very end of the beam (arrow shaped), where solid elements adjacent to the NS must be preserved for the sake of applying the distributed load until the end (recall Figure 3 and Figure 4). After applying the threshold (see Figure 6, top-left), only the domain’s solid part is saved (all void regions are deleted) in CDB file format. The mesh information is preserved, and jagged contours can be recognized. This is an input for ANSYS Workbench^®^, where the FE model can then be saved in an STL format to be exported to Blender^®^ for further processing. Here, ANSYS Workbench^®^ bridges the ANSYS Mechanical APDL^®^ and Blender^®^ environments. Once the optimal structure is imported into Blender^®^, the boundaries are smoothed using the smooth Laplacian modifier (see Figure 6, top-center). This modifier allows for reducing the noise on a mesh while preserving its shape integrity. However, the original right angles must be preserved instead of allowing the smoothing process to round them. This may require additional work in Blender^®^ by creating auxiliary areas and performing trimming. The modifier’s parameters—namely, the “lambda factor”, the “lambda border”, and the “repeat”—take values of 0, 7, and 4, respectively, for the FGM beam example considered in this work. The fine-tuning of these parameters is obviously problem-dependent.

After boundary smoothing, the data are exported from Blender^®^, in STL format, back to ANSYS Workbench^®^, where the 2D structure is reopened, but now as a reshaped area. Only geometric identities (e.g., areas, lines, and keypoints) exist. For comparison with the original optimized structure resulting from TO, this new geometry demands a new FE analysis to evaluate the impact of data conversions in the structural performance. To that end, these data are exported from ANSYS Workbench^®^, in IGS format, back to ANSYS Mechanical APDL^®^, where the new area is meshed using quadrilaterals. This latter mesh (Figure 6, top-right) is typically unstructured, whereas the former (Figure 6, top-left), resulting from TO, is structured. The element centroids’ coordinates on both meshes (see Figure 6, bottom-left and center-right) are saved separately in text files, and they are used for mapping the properties, as detailed below.

**Figure 7:**
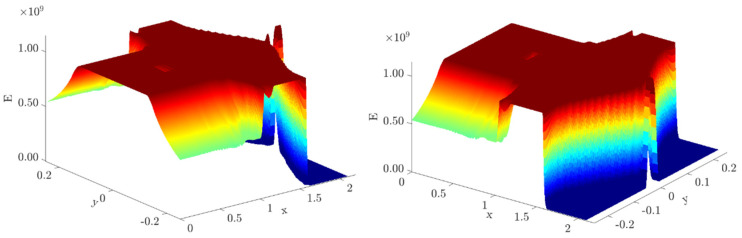
Surface plot of the interpolation function “Surffit1” from two different angles.

Firstly, the original FE centroids’ coordinates are used as sample points’ locations in the “griddedInterpolant” function from MATLAB^®^ (2021 edition). Each element material property, found by FGMTO in the optimization problem formulation stated in Equation (4), is the sample value associated with each sample point, which renders a stepwise property distribution across the domain (see Figure 6, bottom-center). A response surface is then generated using the “Makima” method for both interpolation and extrapolation (see Figure 6, bottom-right). This method is preferred due to its ability to produce a function that mimics the hyperbolic variation in Young’s modulus presented in Section 3.3 and Section 3.4, specifically for the region of the beam that is subject to pure bending, while ensuring a smooth property variation in the remaining regions. The generated interpolation function, resulting from the application of the “griddedInterpolant” function, is called “Surffit1” and retrieves material properties at any query point upon definition of its coordinates (*x*,*y*). Figure 7 plots the corresponding surface “Surffit1”. Using the list of centroids saved at the end of the contour smoothing (Figure 6, center-right) as query points, the property can then be calculated and vectorized in MATLAB^®^ at each and every arbitrarily located element.

Bear in mind that some sample points for “Surffit1” may have nearly zero property values to be interpolated (refer to the void regions in Figure 4). Furthermore, the “Makima” method, using up to third-degree polynomials, may generate unphysical interpolated values once the maximum sample value, $E_{1}$ (recall that $E_{1}>E_{2}$), is exceeded. Therefore, all interpolated/extrapolated property values must be checked and adjusted accordingly, i.e., (1) every value higher than $E_{1}$ is replaced by $E_{1}$ itself, and (2) every value lower than a predefined threshold is replaced by the property value of the nearest element with a property value higher than or equal to the threshold. This threshold should be set to $E_{2}$ for the sake of the materials’ physical meaning, as established in Section 3.1. However, to consistently compare the original results from optimization with those from post-processing data according to the workflow in Figure 6, the property threshold here is set to 0.03439 GPa. This value comes from setting $\rho_{1}=0.5$ (contour-smoothing threshold) and $\rho_{2}=\rho_{min}$ in Equation (1). After the adjustments, a vector containing the material properties at each element’s centroid coordinates in the meshed area that has been smoothed is now saved in a text file format. This is then loaded in ANSYS Mechanical APDL^®^ to set the proper material properties of the FE model. The boundary conditions remain the same between the structured (original) and unstructured meshes. The corresponding FE results can now be compared.

**Figure 8:**
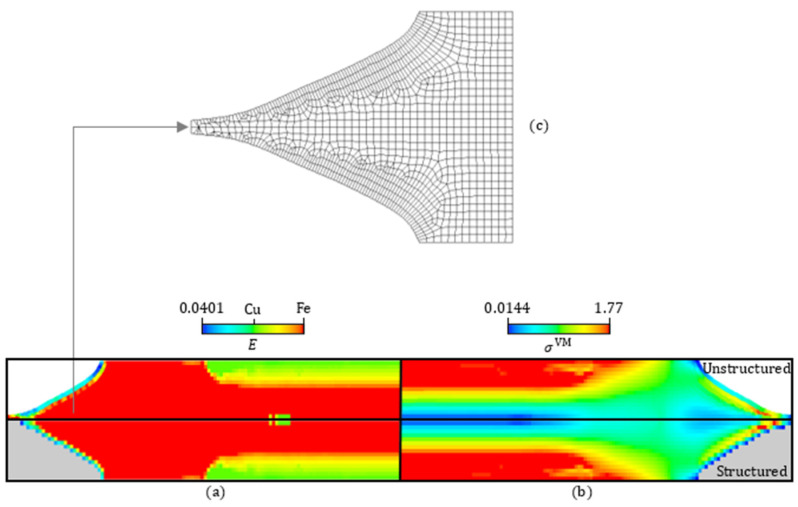
Comparative analysis of results between structured (**top**) and unstructured (**bottom**) meshes for the beam example: (**a**) Young’s modulus distribution [GPa]. (**b**) Von Mises stress map [MPa]. (**c**) Detailed representation of the mesh called Unstructured I.

Taking advantage of the stress and property distributions’ symmetry with respect to both the NS and the mid-span, different results can be presented in quarters of the beam’s domain. Figure 8a,b compare the Young’s modulus distributions and the von Mises stress maps, respectively, between the original (structured) mesh (see bottom) and the unstructured mesh (see top). The color scale is the same between the bottom and top maps. The original mesh elements that were discarded upon thresholding $\rho_{1}$ at 0.5 are colored gray in Figure 8a,b (bottom) to facilitate the comparison. Figure 8c enlarges the unstructured FE mesh, focusing on the beam’s left end. This mesh is denoted here as “Unstructured I”, and it comprises 3635 elements, nearly the same number of elements present in the original square-grid mesh (30 × 126 elements). Although two additional unstructured mesh refinements, called Unstructured II and III (with 6098 and 13,298 elements, respectively), are also considered, only the display of the coarser mesh (Unstructured I) can be comprehensive (see Figure 8c).

**Figure 9:**
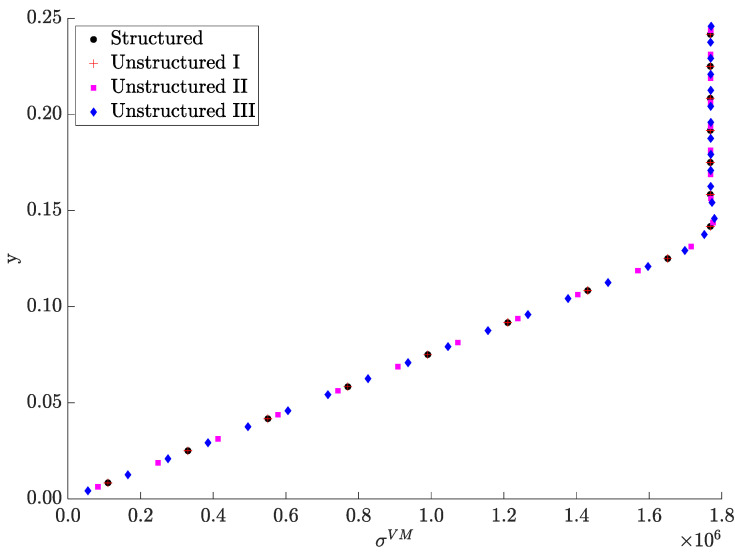
Element centroid coordinate *y* against the respective von Mises stress $\sigma^{VM}$ in the region of the beam subject to pure bending. Stress results are given for the different meshes studied, structured (original) and unstructured (I, II and III).

The comparison established in Figure 8 proves that the Unstructured I mesh, resulting from the proposed workflow, preserves the two main features found at the optimization stage quite well. On the one hand, the lightweight optimal layout obtained from the removal of material leaves each beam’s end in an arrow shape. On the other hand, the layer-wise FGM across the beam’s transverse direction assures a relevant stress reduction. However, regarding this last feature, both meshes (original and Unstructured I) divide the beam’s height into an equal number of FEs. The robustness of the proposed workflow in preserving the benefits of the optimized layer-wise FGM can now be questioned by considering meshes with an arbitrary number of elements. To investigate that, the stress results at the mid-span obtained with the Unstructured II and III meshes are plotted in Figure 9 along with the previous results. The results from the different meshes essentially overlap, following the trend shown in Table 2, as expected. In fact, the highest deviation among all plotted results is only 0.6%. Therefore, this digital data processing workflow looks promising in the light of this beam example shown as a proof-of-concept. The idea is to convert the geometry and property gradient data into an AM-friendly format (e.g., STL file), detached from the TO framework, such that an AM system can easily interpret and reproduce it [89]. However, one must realize that materializing an FGM design requires more than just passing geometry data and optimal constituent volume fractions (see the workflow in Figure 6, right-hand side). It follows that additional WAAM process parameters must be considered. For instance, the predicted volume fraction variations serve as an input for the AM process, rather than the Young’s modulus variations. Section 2.2 presents the curve relating these two factors, but that curve is also sketched in Figure 6. Then, proper trade-offs are required among the voltage, current, wire feed speed, travel speed, and gas flow rate. The fine-tuning of values among these process parameters is invaluable to succeed in printing FGMs, requiring ongoing and future research efforts on its own, and the present framework only paves the way in this regard.

## 7. Discussion

With the advent of AM processes, complex part shapes and a wide range of materials, from commercial alloys to FGMs and composites, can now be fabricated. Design methods, such as TO, typically result in design solutions that are among the most efficient ones. Therefore, a natural symbiosis exists between these two emerging technologies (i.e., AM and TO) [41]. In addition to using multiple materials in TO, FGMs have also started to be explored in TO [90,91]. Conventional experimental apparatus used in uniaxial tensile tests for assessing mechanical properties and responses can be quite limited, often failing to capture the increased level of detail when a compositional gradient exists. Modern methods based on full-field analysis, such as DIC, fully explore the range of data that can be measured from such complex parts [92]. In polymer applications, the pioneering work of Ituarte et al. [25] has closed an engineering cycle (TO → AM → DIC), successfully producing FGM parts as a proof-of-concept. This shows that great achievements have already taken place in polymers, mainly due to the rapid development of dedicated AM methods, allowing for refined printing resolutions, and the simpler processing of polymer mixtures compared to metals. Building on this innovative design concept, the present work encourages further exploration of a similar approach, with a focus on metal-based FGMs and structures, covering the entire process cycle: TO → WAAM → DIC. To the best of the authors’ knowledge, no published works have yet fully completed such a cycle for metals. The few contributions available in the literature explore trends and future outlooks [24].

To start with, the present work features possible metal–metal binary systems, realizing that their success in producing FGMs is largely constrained by their metallurgical compatibility (e.g., the formation of solid solutions or brittle intermetallics) and weldability/printability problems (e.g., susceptibility to hot cracking). The theoretical critical analysis pursued in Section 2.1 needs to be supplemented with experimental evaluations across a broader range of compositions, which have not yet been much explored, as perceived in the critical review work by Reichardt et al. [26]. These authors also provided a cross-comparison of various classes of metals (see also [93]). Based on these critical analyses, a few binary systems, such as Cu-Fe, Cu-Ni, or Fe-Ni, appear more promising, as commonly observed in their welded joints [94,95]. In addition, it is worth mentioning that FGMs can also be manufactured using feedstock materials from the same alloy class (or family), such as FGMs produced from low-alloy and stainless steels, or those fabricated using only stainless steels (e.g., martensitic and austenitic [96]). As discussed in Section 4, the present work identifies the double-wire-feed WAAM variant (GTAW-based process) as the most suitable technology for producing FGM structural parts, as it allows the use of different feedstock materials in a predefined prescription of volume fractions, enabling precise tailoring of the microstructure and chemical composition along the deposition directions.

In the absence of experimental data for all alloy combinations presented in Table 1, theoretical predictions, such as the HS bounds, have been used for optimal FGM designs, as seen in the pioneering work of Xia and Wang [29]. The present work, in Section 2.2 and Section 3.1, recovers this theoretical approach by assuming isotropy of solid mixtures, which may be a limiting hypothesis. In fact, this material symmetry has not been verified for parts manufactured using the WAAM process, as reported in the literature [60,97,98,99,100], which is associated with the coarse and oriented microstructure typically observed in parts fabricated by low-energy-density AM processes such as arc-based directed energy deposition. This is an important consideration for future developments of this work, especially since different binary systems may exhibit varying levels of orthotropy.

So far, FGMs have been pointed out as effective solutions for stress mitigation. However, this claim is often based on analyses rather than optimization works [101,102,103,104]. Using TO in this work, we fully explored the design freedom; thus, the potential of FGMs comes even more to the fore. To demonstrate this, we resorted to an FGM beam design problem. Typically, predefined gradients, such as power and exponential laws [77,78], are used in beam studies, but this limits the design space. Reformulating the FGM design problem using optimization methods reveals the real benefits of continuous compositional grading, achieving a peak stress reduction of up to 26% in the beam problem addressed in this work as a proof-of-concept. The optimal hyperbolic unidirectional gradient found numerically for this beam example is thus to be interpreted as functional in load-bearing structures. This hyperbolic gradient, analytically validated here for the first time, differs from previous studies that essentially relied on predefined gradient laws [78].

The conceptual FGM design obtained by density-based TO (pixelized) must be converted to the AM system, addressing not only contour smoothing but also the mapping of material properties. While this combined challenge has been raised in previous reviews [21,89], it remains noticeably understudied. The proposed digital workflow in Section 6 offers a valuable and inspiring tool for bridging the gap between design and fabrication in metal AM for FGMs, as validated using the beam example proposed here. Although continuous compositional grading can be achieved in silico, printing it layer by layer is unlikely due to the resolution limitations of the AM processes. In practice, the chemical composition of the melt pool, even in mixed materials like FGMs or dissimilar welding, is typically considered to be macroscopically homogeneous [105,106], even in unidirectionally fabricated single-bead multi-layer parts (e.g., simple walls). Future experiments are needed to provide feedback to computational models, focusing not only on the directionality of properties but also on the thickness and resolution of printed layers.

## 8. Conclusions and Future Trends

This work explores the manufacturing of metal-based FGMs within the framework of an AM engineering cycle, combining TO and WAAM. Given the better performance of metallic structures compared to ceramics (brittle and heavy) and polymers (low strength and compliance), this research focuses on minimizing stresses within these structures by utilizing FGMs. This enlarges the design space, allowing for improved structural performance. Furthermore, the present work also raises awareness that metal–metal binary systems, in the context of metal AM for creating FGMs, bring important metallurgical concerns that can manifest during the printing of parts, such as hot cracks and the formation of intermetallic compounds. This highlights the complexities involved in materializing optimal design solutions that combine dissimilar metals. In fact, most combinations of metallic materials do not exhibit full solubility across the complete range of constituent volume fractions. In short, this study provides a theoretical critical analysis, using phase diagrams of pure metals, to evaluate the advantages and disadvantages of various metal–metal binary systems for use in FGM design solutions. Practical research in materials science and fabrication technologies is essential to determine the feasibility of specific combinations. The Fe-Cu system was selected in this work to generate a computational FGM optimal design as a proof-of-concept.

The type of FGM addressed in this work involves gradual changes in the volume fractions of the constituent materials, such that the resulting heterogeneous structures provide continuous-graded macroscopic properties. Unlike previous research that resorted to predefined property gradient models, e.g., exponential or power law models, the present framework aims to find the optimal property gradient across the structure’s domain, thus avoiding any a priori gradient assumption. This approach is based on the efficient use of material resources, which matches the concept of TO. The design approach proposed here adequately predicts the property gradient to excel in structural performance. In the beam example presented in this work, the related pure bending stresses are minimized on account of an FGM, where the variation in Young’s modulus across the beam’s cross-section is given by a hyperbolic law. This compositional gradation comes along with optimal layout changes to fully explore the design freedom. Also noteworthy is the fact that the aforementioned hyperbolic trend remains optimal for any property (Young’s modulus) ratio other than the pre-selected one for the Fe-Cu system, as proven here by using an analytical beam model considering pure bending.

The close bond between TO and AM is the key to materializing FGM designs. Although this duo has matured with regard to single- and multi-material solutions, in the context of FGMs there is still a long way to go to complete the AM engineering cycle, going from in silico to produced parts, followed by characterization. The interface between design and fabrication has typically involved Laplacian smoothing, ending up with STL files containing the geometry for 3D printing. The data transfer becomes more complex when AM systems must reproduce not only geometry but also property gradients, which has barely been addressed in the literature. The workflow presented in this work is a step towards the completion of FGMs’ realization by performing data conversion pertaining not only to geometry but also to property gradients. Density-based TO is based upon an original meshed (pixelized) domain that is not adequate for fabrication. The several steps of the proposed workflow aim to ultimately handle mesh data and property gradients independently of the original domain discretization, i.e., considering any arbitrary meshed domain (unstructured meshes) of any resolution. The procedure was tested using an FGM beam example, and it proved to be effective in preserving valuable design data from the original optimal design result. Success in materializing such FGM parts demands further research, particularly in optimizing the fabrication process parameters of the AM system, which typically require fine-tuning. Also, the printing’s spatial resolution, as well as the compositional gradation rates, can be limited by the AM system. Once the part is produced, valuable image-based characterization techniques, such as DIC, can help validate or adjust the initial numerical design model based on the measured properties and the limitations introduced by the fabrication process. Therefore, the data flow among TO, AM, and DIC plays an important role in realizing metallic graded structures, and much further research work is demanded ahead.

In the near future, the fabrication of specimens with different compositions by joining dissimilar metals is envisaged, exploring their full (or quasi-full) compatibility and solubility across the range of constituents’ volume fractions. Upon proper characterization of these specimens, computational material models can be enhanced and used to predict the optimal gradations to excel in performance. The layer-wise FGM concept, as explored in this framework, can be a promising start for reproducibility in metal AM systems, although, to gain insights into process parameters, simpler predefined gradients (e.g., linear gradients) are most likely advisable to start with.

## Figures and Tables

**Table 1:** Metal–metal binary systems shown in matrix format. Pure metals are selected, and their respective non-oriented Young’s modulus values are indicated in GPa. The upper triangular portion shows the Young’s modulus ratios ($E_{2}$/$E_{1}$ with $E_{1}>E_{2}$). The lower triangular portion highlights the main challenges related to printing each metal–metal pair. A dilution level between 10% and 30%, typically observed in DED processes, was selected. I, H, and G denote susceptibility to intermetallic formation, susceptibility to hot cracking, and the potential suitability of the binary system to be a good candidate, respectively. Moreover, thermodynamic data are unavailable for certain conditions (–).

Material	*Molybdenum*	*Chromium*	*Cobalt*	*Nickel*	*Iron*	*Vanadium*	*Titanium*	*Copper*	*Niobium*	*Zirconium*	*Aluminum*	*Magnesium*
Abbrev. *E* [GPa]	Mo 330	Cr 248	Co 211	Ni 207	Fe 200	V 126	Ti 116	Cu 110	Nb 103	Zr 95	Al 68	Mg 44
Mg	0.133	0.177	0.209	0.213	0.220	0.351	0.379	0.400	0.427	0.466	0.647	
Al	0.206	0.274	0.322	0.329	0.340	0.542	0.586	0.618	0.660	0.720		I
Zr	0.286	0.381	0.448	0.457	0.473	0.753	0.815	0.859	0.917		I	–
Nb	0.312	0.415	0.488	0.498	0.515	0.821	0.888	0.936		G	I/H	–
Cu	0.333	0.444	0.521	0.531	0.550	0.876	0.948		G/H	I	H/I	H/I
Ti	0.352	0.468	0.550	0.560	0.580	0.924		I	G	G	I	–
V	0.380	0.506	0.595	0.606	0.628		G	G	G	G	I	–
Fe	0.606	0.806	0.948	0.966		I	I	G/H	I	I	I	I
Ni	0.627	0.835	0.981		G/H	I	I	G	I/H	I	I	I
Co	0.639	0.851		G	G	I	I	G	I	–	I	–
Cr	0.752		I	G	G	–	I	G	H	H	I	H
Mo		G	I	G	G	–	G	–	G	–	I	–

**Table 2:** Optimal variation in the Young’s modulus E(y), and respective normal stress distribution $\sigma_{xx}\left(y\right)$, applied to an FGM beam under pure bending.

y∈	$E\left(y\right)$	$\sigma_{xx}\left(y\right)$	$E\left(y\right)$$\;\mathrm{and}\;\sigma_{xx}\left(y\right)$ Plots
$\left[+\hat{r}\frac{h}{2},+\frac{h}{2}\right]$	$\frac{{hE}_{2}}{2y}$	$\frac{1}{h^{2}}\left(\frac{12M}{3-{\hat{r}}^{2}}\right)$	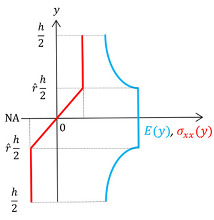
$\left[-\hat{r}\frac{h}{2},+\hat{r}\frac{h}{2}\right]$	$E_{1}$	$\frac{0.5y}{{\hat{r}h}^{3}}\left(\frac{12M}{3-{\hat{r}}^{2}}\right)$	
$\left[-\frac{h}{2},-\hat{r}\frac{h}{2}\right]$	$-\frac{{hE}_{2}}{2y}$	$-\frac{1}{h^{2}}\left(\frac{12M}{3-{\hat{r}}^{2}}\right)$	

## Data Availability

The raw data supporting the conclusions of this article will be made available by the authors on request.

## Abbreviations

The following abbreviations are used in this manuscript:

AM	Additive Manufacturing
DIC	Digital Image Correlation
DfAM	Design for Additive Manufacturing
FEM	Finite Element Method
FE	Finite Element
FGAM	Functionally Graded Additive Manufacturing
FGM	Functionally Graded Material
FGMTO	Functionally Graded Material Topology Optimization
GMAW	Gas Metal Arc Welding
GTAW	Gas Tungsten Arc Welding
HS	Hashin–Shtrikman
MMA	Method of Moving Asymptotes
NA	Neutral Axis
NS	Neutral Surface
RAMP	Rational Approximation of Material Properties
SIMP	Solid Isotropic Material with Penalization
TO	Topology Optimization
WAAM	Wire-Arc Additive Manufacturing
